# TRAIL-R1 as a novel surface marker for circulating giant cell tumor of bone

**DOI:** 10.18632/oncotarget.17042

**Published:** 2017-04-11

**Authors:** Jian-Xiang Liu, Zhi-Cai Zhang, Zeng-Wu Shao, Fei-Fei Pu, Bai-Chuan Wang, Yu-Kun Zhang, Xian-Lin Zeng, Xiao-Dong Guo, Shu-Hua Yang, Tong-Chuan He

**Affiliations:** ^1^ Department of Orthopedics, Union Hospital, Tongji Medical College, Huazhong University of Science and Technology, Wuhan 430022, China; ^2^ Molecular Oncology Laboratory, Department of Orthopaedic Surgery, The University of Chicago Medical Center, Chicago 60637, IL, USA

**Keywords:** circulating tumor cells (CTCs), giant cell tumor of bone (GCT), TRAIL-R1

## Abstract

Giant cell tumor of bone (GCT), which frequently occurs in the patients’ spine, is relatively prevalent in Chinese population. A group of GCT invades into vessels and appears to be circulating tumor cells (CTCs) responsible for the distal metastasis of the primary tumor. So far the cell surface markers of GCT have not been determined. In the current study, we aimed to identify a novel CTC marker with higher specificity in GCT. TRAIL-R1+ cells were purified from GCT cell lines. The TRAIL-R1+ cells were compared with total GCT cells for tumor sphere formation, chemo-resistance, tumor formation in nude mice, and frequency of developing distal metastases. We found that TRAIL-R1+ GCT cells appeared to be highly enriched for CTCs in GCT. Compared to total GCT cells, TRAIL-R1+ GCT cells generated significantly more tumor spheres in culture, were higher chemo-resistant, and had a higher frequency of being detected in the circulation after subcutaneous transplantation as well as development of distal metastases. Thus, we conclude that TRAIL-R1+ may be a novel CTC marker in GCT. Selective elimination of TRAIL-R1+ GCT cells may improve the current GCT therapy.

## INTRODUCTION

Giant cell tumors of bone (GCT) are rare bone tumors worldwide, but relatively prevalent in Chinese population [1]. Most GCT appear to be benign connective tissue neoplasms, which consist of osteoclast-like giant cells, stromal cells, and tumor associated monocytes/macrophages [2]. The stromal cells represent the neoplastic component of the tumor, due to their capacity of propagation, proliferation and invasion to peripheral tissue [3].

Chemotherapy is an important method to assist radiotherapy for treating GCT [4]. Denosumab is a novel and effective treatment for aggressive and recurrent GCT [5, 6]. Since not all GCT cells are sensitive to denosumab, it is important to figure out the mechanisms underlying the chemo-resistance of these GCT cells [7].

During pathological progression of GCT, a group of GCT cells may invade into vessels and become circulating tumor cells (CTCs) that had dissembled from the primary tumor and subsequently invaded into the blood circulation [8]. The most critical CTCs should be those have the capacity to form distal tumor, and this fraction of CTCs share characteristics of cancer stem cells [9–11]. Certain type of CTCs could be enriched with specific surface biomarkers, such as CD133 and CD44 [12]. However, no surface markers for CTCs in GCT have been determined.

Activation of the TNF-related apoptosis-inducing ligand (TRAIL) pathway regulates cancerous cell apoptosis in human. TRAIL-R1 is a receptor for apoptosis ligand TRAIL. Very recently, TRAIL-R1 has been reported to play a role in the carcinogenesis [13–15]. However, TRAIL-R1 has not been used as a CTC surface marker in any types of cancer. Here, we studied TRAIL-R1 as a CTC marker for GCT.

## RESULTS

### Labeling of GCT cells with GFP and luciferase

**Figure 1:**
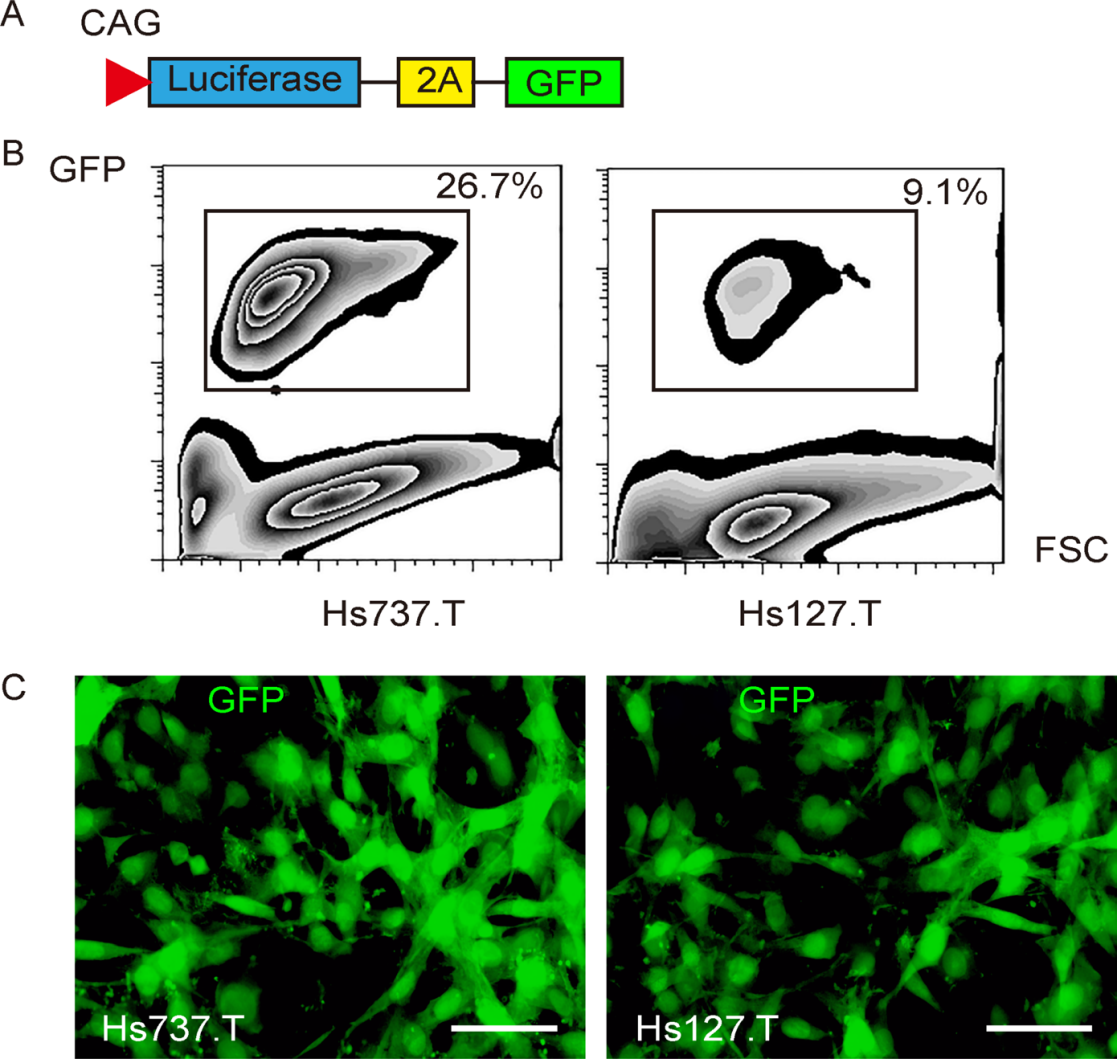
Labeling GCT cells with GFP and luciferase (**A**) To allow tracing tumor formation in living mice and isolation of tumor cells from mice, we transduced the GCT cells with a lentivirus carrying both luciferase and GFP reporter under the control of a CAG promoter. The viral structure is shown. (**B**) The transduced GCT cells were purified based on GFP expression by flow cytometry. (**C**) The purified GFP+ GCT cells in culture. Scale bar is 20 μm.

Two human GCT cell lines Hs737.T and Hs127.T was used in the current study. Both lines were generated from giant cell sarcoma from the bone. A lentivirus carrying both luciferase and GFP reporter under the control of a CAG promoter was used to transduce Hs737.T and Hs127.T cells, for tracing tumor formation in living mice and for isolating tumor cells from mice (Figure 1A). After transduction, the GFP+ GCT cells were purified with flow cytometry (Figure 1B), and appeared to be green fluorescent under fluorescence microscopy (Figure 1C).

### Isolation of TRAIL-R1+ GCT cells

**Figure 2:**
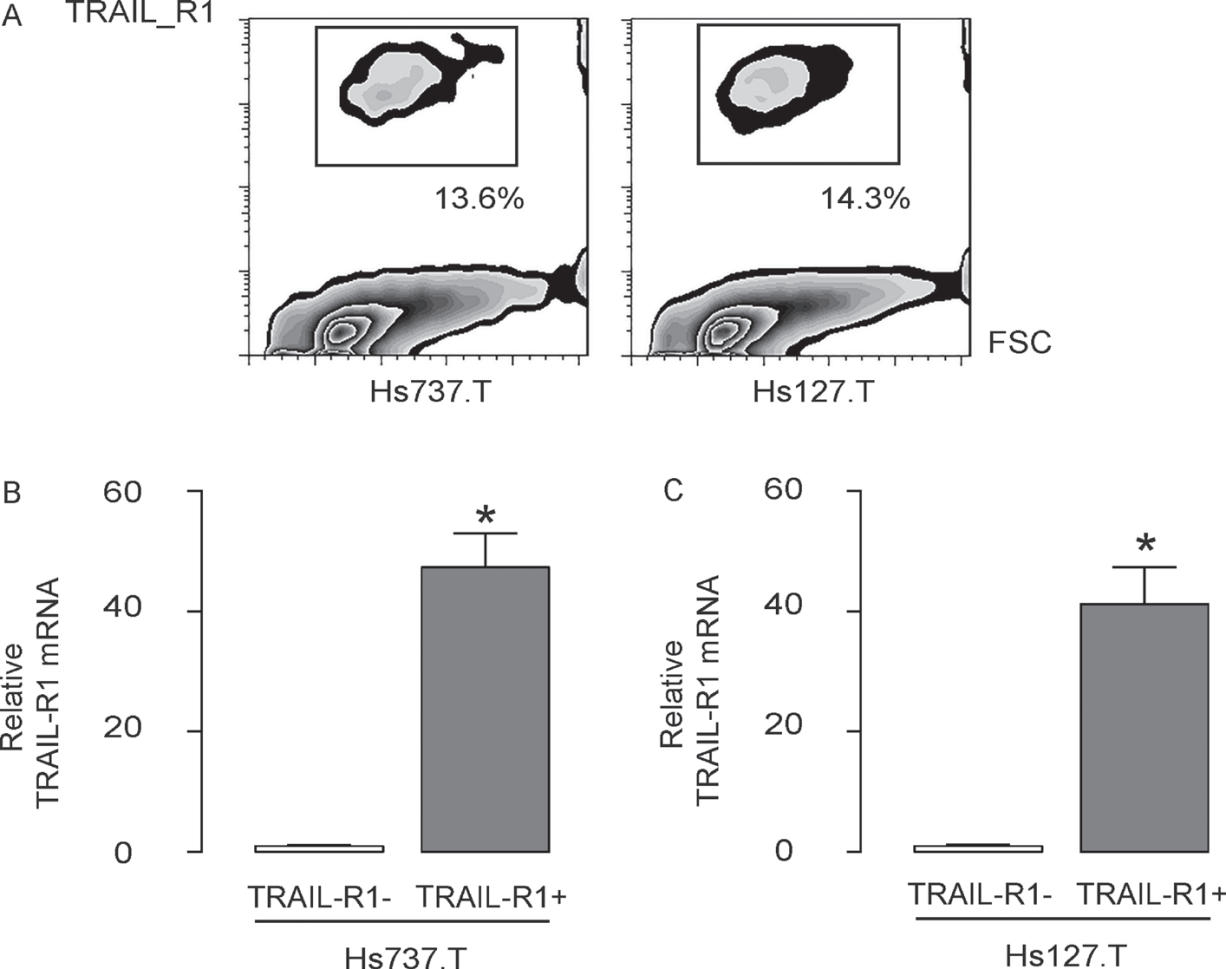
Isolation of TRAIL-R1+ GCT cells (**A**) TRAIL-R1 was used to separate GCT cell population into 2 fractions, TRAIL-R1+ and TRAIL-R1- cells, by flow cytometry. (**B**) RT-qPCR for TRAIL-R1 levels in the TRAIL-R1+ cells and TRAIL-R1- cells. *p < 0.05. *N* = 5.

TRAIL-R1 was used to separate GCT cell population into 2 fractions, TRAIL-R1+ and TRAIL-R1- cells, by flow cytometry (Figure 2A). We checked the quality of purification by examined the TRAIL-R1 levels in the sorted cells by RT-qPCR, showing more than 40 times higher TRAIL-R1 levels in the TRAIL-R1+ cells, compared to TRAIL-R1- cells, in both lines (Figure 2B–2C).

### TRAIL-R1+ GCT cells generate more tumor spheres and are more chemo-resistant *in vitro*

**Figure 3:**
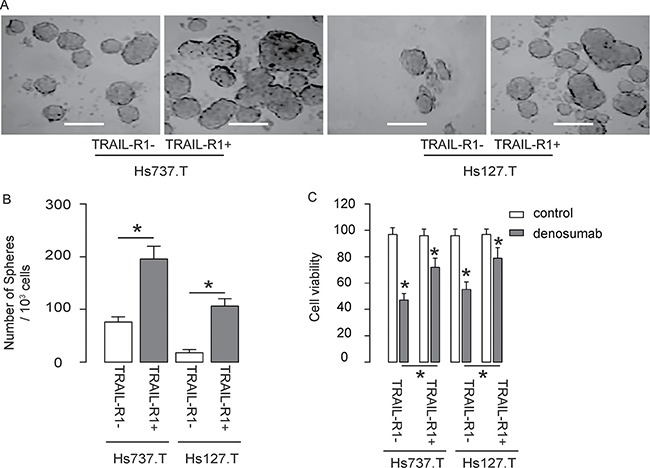
TRAIL-R1+ GCT cells generate more tumor spheres and are more chemo-resistant *in vitro* (**A**–**B**) Tumor sphere formation assay for TRAIL-R1+ and TRAIL-R1- cells, shown by quantification (**A**), and by representative images (**B**). (**C**) TRAIL-R1+ and TRAIL-R1- cells were subjected to denosumab *in vitro*. The cell viability was determined in an CCK-8 assay. *p < 0.05. *N* = 5.

The TRAIL-R1- and TRAIL-R1+ cells from both lines were then subjected to tumor sphere formation assay. We found that compared to TRAIL-R1- cells, TRAIL-R1+ cells generated significantly more tumor spheres in both lines, shown by representative images (Figure 3A), and by quantification (Figure 3B). Next, the TRAIL-R1- and TRAIL-R1+ cells were exposed to denosumab, an effective chemotherapeutic treatment for GCT. We found that compared to TRAIL-R1- cells, TRAIL-R1+ cells appeared to be more resistant to denosumab, since higher cell viability was detected in TRAIL-R1+ cells in either line in an CCK-8 assay (Figure 3C). Hence, TRAIL-R1+ GCT cells generate more tumor spheres and are more chemo-resistant *in vitro*.

### Transplanted TRAIL-R1+ GCT cells generate bigger tumor and are more frequently detected in the circulation

**Figure 4:**
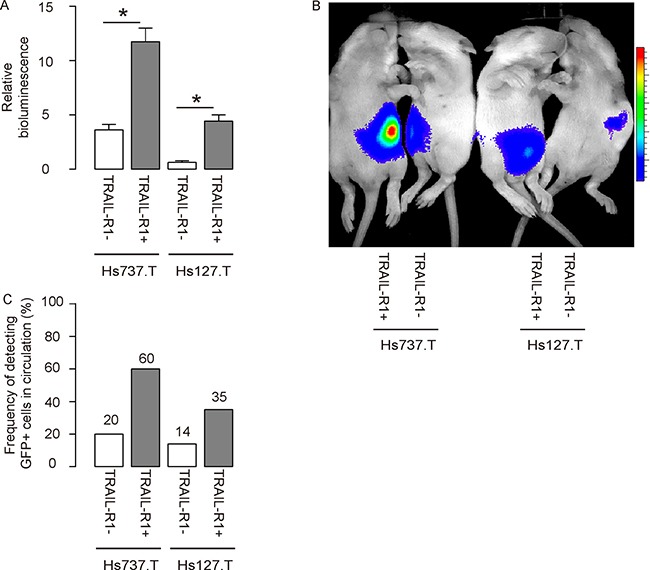
Transplanted TRAIL-R1+ GCT cells generate bigger tumor and are more frequently detected in the circulation Same number of TRAIL-R1- and TRAIL-R1+ cells was subcutaneous transplanted into Nude mice, and the tumor formation was monitored after 8 weeks. (**A**–**B**) The luciferase activity was determined in mice, shown by quantification (**A**) and by representative images (**B**). (**C**) A total of 100 μl of mouse blood was taken for detection of GFP+ cells by flow cytometry. The frequency was shown. *p < 0.05. *N* = 30.

Same number of TRAIL-R1- and TRAIL-R1+ cells was subcutaneously transplanted into Nude mice, and the tumor formation was monitored after 8 weeks. We found that compared to TRAIL-R1- cells, TRAIL-R1+ cells generated significantly larger tumor shown by quantification (Figure 4A) and by the representative images for bioluminescent examination (Figure 4B). In order to check whether TRAIL-R1+ cells may be enriched for CTCs, we examined the mouse blood at 8 weeks and we found that green tumor cells were more frequently detected in the circulation of mice transplanted with TRAIL-R1+ GCT cells (Figure 4C).

### Higher occurrence of tumor formation was detected after serial adoptive transplantation of TRAIL-R1+ GCT cells

**Figure 5:**
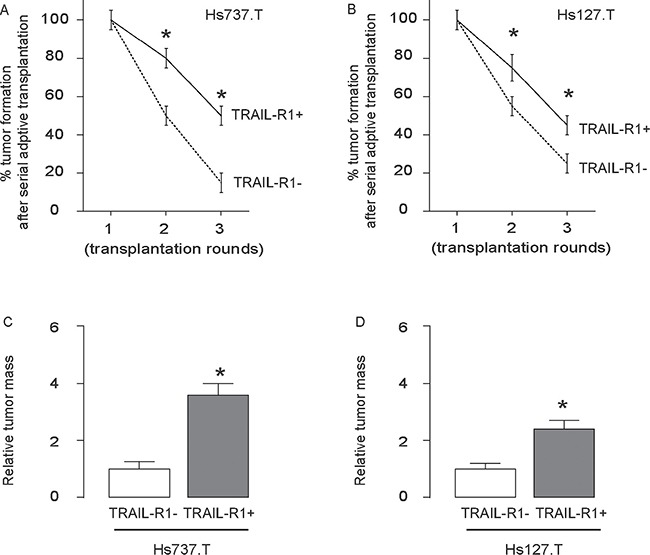
The highest occurrence of tumor formation was detected after serial adoptive transplantation of TRAIL-R1+ GCT cells (**A**–**B**) 30 GCT cells were isolated from the GCT tumor developed from TRAIL-R1- or TRAIL-R1+ cells, and were transplanted back to new nude mice. The formation of the tumor was first verified by bioluminescence and then confirmed by histology of the dissected out at 6 weeks. The newly formed tumors were then dissected out and used for isolation of 30 tumor cells for the next round of transplantation. Three rounds of transplantation were performed. (**A–B**) Frequency of developing tumor by Hs737.T cells (**A**) and by Hs127.T cells (**B**). (**C**–**D**) The relative tumor mass by Hs737.T cells (C) and by Hs127.T cells (D). *p < 0.05. *N* = 30.

Finally, 30 tumor cells were isolated from the primary tumor developed from either TRAIL-R1- and TRAIL-R1+ cells, and were transplanted back to new nude mice. The formation of the tumor was first verified by bioluminescence and then confirmed by histology of the dissected out at 6 weeks. The newly formed tumors were then dissected out and used for isolation of 30 tumor cells for the next round of transplantation. Three rounds of transplantation were performed. We found that TRAIL-R1+ cells had significantly higher rate of tumor formation after serial adoptive transplantations, compared to TRAIL-R1- cells, based on bioluminescence examination (Figure 5A–5B). Moreover, the tumor mass formed by TRAIL-R1+ cells was significantly greater, compared to TRAIL-R1- cells (Figure 5C–5D).

## DISCUSSION

In the current study, we analyzed TRAIL-R1 as a novel CTC marker for GCT. The ignition of the study was encouraged by a recent study in neural science, which shows that TRAIL receptors may play a role in the radio-resistance of human neural stem cells and neuroblastoma cells [16]. We have analyzed other members from TRAIL family and only got positive results from TRAIL-R1.

We chose two human GCT lines in this study, since both represent giant cell tumor of the bone, but appear to be different in malignancy. Hs737.T was from a 10-year-old female, while Hs127.T was from a 64-year-old male. Hs737.T grew faster and appeared to be more aggressive than Hs127.T in culture. Thus, analysis on both lines increased the reliability of the study and the results may be more applicable to GCT.

We purified TRAIL-R1+ GCT cells, and compared to TRAIL-R1- GCT cells. We found that these TRAIL-R1+ cells showed higher tumor formation potential *in vitro* and *in vivo*, appeared to be more resistant to denosumab, were more frequently detected in the circulation, and generated tumor more frequently after serial adoptive transplantation. These are gold standards for determining CTC with tumor forming potentials. Indeed, here we used a set of experiments to characterize that TRAIL-R1+ GCT cells may be a fraction of circulating tumor stem-like cells (CTSC), which have properties of tumor formation, high invasiveness and chemo-resistance [17]. Identification of such a population in GCT has great clinic importance.

Denosumab is a FDA-approved human monoclonal antibody with high affinity and specificity to RANKL, the receptor activator of nuclear factor kappaB ligand, which is the principal mediator of osteoclastic bone resorption [5, 6]. The initial study on denosumab has focused on its effective suppression of bone resorption in a rapid, sustained, but reversible manner [5, 6]. However, the well-known feature of GCT to express RANKL has invited many studies of denosumab on GCT treatment and shown demonstrative effects. Since the effects of denosumab are not consistent on all GCTs, here we present a possible explanation that a fraction of GCT population, CTCs, may be more resistant to denosumab treatment. Future studies may be applied to analyze the RNAKL expression in populations expressing different level of TRAIL-R1. The study of the underlying molecular pathway may be interesting for understanding the pathogenesis and tumorigenesis of GCT.

Although here we provide direct data to support the usefulness of TRAIL-R1 as a surface marker for enriching CTSC in GCT, further evidence from clinical studies is required to confirm the findings. Nevertheless, here our study demonstrates that TRAIL-R1+ may be a novel CTC marker in GCT. Selective elimination of TRAIL-R1+ GCT cells may improve the current GCT therapy.

## MATERIALS AND METHODS

### Protocol approval

All the experimental methods have been approved by the research committee at Union Hospital, Tongji Medical College. All animal experiments were approved by the Institutional Animal Care and Use Committee at Union Hospital, Tongji Medical College. All the experiments have been carried out in accordance with the guidelines from the research committee at Union Hospital, Tongji Medical College. The methods regarding animals were performed under the approval of institutional Ethics Committee.

### Cell culture and treatment

Two human GCT cell lines Hs737.T and Hs127.T were both purchased from ATCC (American Type Culture Collection, Manassas, VA, USA). These cells were cultured in Dulbecco's Modified Eagle's Medium suppled with 10% Fetal Bovine Serum (FBS, Sigma-Aldrich, San Jose, CA, USA). The cells were incubated in a 37°C incubator with 5% CO_2_ Denosumab (XGEVA, Amen, Thousand Oaks, CA, USA) was given to cultured cells at the concentration of 10 μmol/l. The cells were analyzed 24 hours after treatment.

### Cell transduction

The GCT cells transduced with lentivirus carrying Luciferase (LUC) and green fluorescent protein (GFP) reporters for *in vivo* visualization of the implanted tumor cells and for detection of the CTCs by flow cytometry. The pcDNA3.1-CAG-GFP plasmid and a pcDNA3.1-CAG-luciferase plasmid were applied in backbones (Clontech, Mountain View, CA, USA). The GFP coding sequence was digested with Xhol and BamHI and subcloned with a 2A into a pcDNA3.1-CAG-luciferase, and pCAG-luciferase-2A-GFP was obtained. For constructing lentiviral particles, HEK293T cells were seeded in 100 mm dish at 50,000 cells/cm^2^ and co-transfected with 10 μg of pCAG-luciferase-2A-GFP and 5 μg each of packaging plasmids (REV, pMDL and VSV-G) with Lipofectamine-2000 (Invitrogen). The supernatant was removed 48 hours after transfection and filtered through the 0.45 μm syringe filter. The virus in supernatant was isolated and titered. For cell transduction *in vitro*, GCT cells were seeded in 100 mm plates at 15,000 cells/cm^2^ one day prior to lentiviral infection. The lentiviral particles were added along with 10 μg/ml polybrene (Sigma-Aldrich) to the cell culture at a multiplicity of infection (MOI) of 100 for 48 hours. Then the cells were rinsed twice with complete media and purified for transduced cells (efficiency of 26.7% for Hs737.T and of 9.1% for Hs127.T cells) based on GFP with flow cytometry. The transduced cells were observed *in vivo* with luciferase, and determined with GFP.

### Animal manipulation

Ten week-old male NOD/SCID mice (SLAC Laboratory Animal Co. Ltd, Shanghai, China) were used for subcutaneous transplantation of tumor cells and serial adoptive transfer. The bioluminescence was monitored 4 weeks after transplantation. For transplantation of cancerous cells into NOD/SCID mice, 200 cells were implanted and the tumor formation was monitored after 8 weeks by bioluminescence. For serial adoptive transplantation of cancer cells, 30 cancer cells were isolated from implanted tumor and re-transplanted back into the mice. The tumor formation was examined after 6 weeks by bioluminescence. Three rounds of serial adoptive transfer were performed.

### Tumor monitoring by bioluminescence

Formation of tumor was monitored by luciferin assay, based on luciferase activity of tumor cells. All the mice were anesthetized with 3% isoflurane and then luciferin (Sigma-Aldrich) of 150 mg/kg body weight were injected intraperitoneally. After 10 minutes, the bioluminescence of mices were observed and imaged with IVIS imaging system (Xenogen Corp., Alameda, CA, USA), with an acquisition time of 60-second and binning of 10. The images were processed and analyzed with the software of Living Imaging system.

### Primary tumor sphere culture

After cancer cells were obtained, all the cells were dispersed to single cells with enzymatic digestion. Afterward, single cancer cells were re-suspended in tumor sphere media (TSM). The TSM was a serum-free DMEM, with human recombinant Epidermal growth factor (20 ng/ml), bFGF (20 ng/ml), leukemia inhibitory factor (10 ng/ml) and N-acetylcysteine (60 μg/ml). The cells were then smeared in 60 mm petri dish at a density of 2 × 10^4^ cells/plate. Then the formation of tumor sphere was observed and recorded.

### Cell viability assay

The cell viability was determined with CCK-8 detection kit (Sigma-Aldrich). First, cells were prepared with a density of 5 × 10^4^/ml and seeded in a 96-well microplate. After 24 h, cells were treated with resveratrol. Then the CCK-8 reagents were added and incubated. The absorbance of wells in microplate was read at 450 nm with microplate reader. The absorbance value was positively correlated to cellular viability. The cell viability was calculated as: the percentage of absorbance value in detected well with reference to control well (control group without treatment was 100%).

### Statistical analysis

The statistical analysis was performed with the GraphPad Prism 6 (GraphPad Software, San Diego, CA, USA). Comparison of group differences was carried out with a one-way analysis of variance (ANOVA) test and then Turkey multiple comparison post-hoc analysis. All values represent the mean ± standard deviation (SD). A value of *p* < 0.05 was considered as significant after Bonferroni correction.
